# EZ-DripLoss Assessment in Chicken Breast Meat Using Different Sample Areas, Fiber Orientation, and Measurement Intervals

**DOI:** 10.3390/ani11041095

**Published:** 2021-04-12

**Authors:** Ana Kaić, Zlatko Janječić, Andrija Žanetić, Nikolina Kelava Ugarković, Klemen Potočnik

**Affiliations:** 1 Department of Animal Science and Technology, University of Zagreb Faculty of Agriculture, Svetošimunska Cesta 25, 10002 Zagreb, Croatia; andrija.7595@hotmail.com (A.Ž.); nkelava@agr.hr (N.K.U.); 2 Department of Animal Nutrition, University of Zagreb Faculty of Agriculture, Svetošimunska Cesta 25, 10002 Zagreb, Croatia; zjanjecic@agr.hr; 3 Department of Animal Science, Biotechnical Faculty, University of Ljubljana, Groblje 3, SI-1230 Domžale, Slovenia; klemen.potocnik@bf.uni-lj.si

**Keywords:** core diameter, holding time, meat quality, myofibril direction, poultry, water-holding capacity

## Abstract

**Simple Summary:**

Drip loss is one of the most important parameters of meat quality evaluation. A new method for the determination of drip loss in poultry meat is the EZ-DripLoss method. The results of the drip loss method used are highly dependent on the surface area, its weight, the fiber direction of the sample, and the storage period. From the literature reports it is obvious that there is a deficiency in our knowledge of the EZ-DripLoss method’s suitability for poultry meat analysis. Therefore, this study aimed to research the effect of different sample core diameters (10, 20, and 30 mm), and fiber orientations (vertical vs. horizontal) on the EZ-DripLoss in chicken breast meat measured across a period of five days. The results indicate that the EZ-DripLoss for chicken breast meat is highly dependent on the sample area, fiber orientation, and measurement interval. Although the EZ-DripLoss method is considered to be simple and reproducible, it is still insufficiently researched and standardized. Comparison of the EZ-DripLoss results obtained with different sampling procedures should be performed with great precaution.

**Abstract:**

Although the EZ-DripLoss method has been performed in numerous studies, there is a deficiency in our knowledge of the EZ-DripLoss method’s suitability for poultry meat analysis. This study aimed to research the effect of different sample areas (10, 20, and 30 mm; *n* = 240), and fiber orientations (vertical vs. horizontal) on the EZ-DripLoss in chicken breast meat measured across a period of five days. The influence of sample area on the EZ-DripLoss of chicken breast meat with respect to the fiber orientation and across the five-day measurement interval was significant between 10 and 30 mm samples, and between 20 and 30 mm samples (*p* < 0.001). The estimated regression coefficient showed that EZ-DripLoss for the samples with horizontal and vertical fiber direction of 10 and 20 mm, significantly increased by 0.04% per hour, while for the samples with vertical fiber direction whose diameter was 30 mm, it significantly increased by 0.06% per hour, and for the horizontal fiber direction, it significantly increased by 0.07% per hour. The samples with vertical fiber orientation had 0.50% greater EZ-DripLoss compared to the samples with horizontal fiber orientation. EZ-DripLoss evaluation in chicken breast should be performed with a sample core diameter of 20 mm, a vertical fiber orientation, and over the course of longer measurement intervals.

## 1. Introduction

The quality of poultry meat relates largely to its water-holding capacity (WHC) which is associated with processing technology and consumer acceptance. WHC in the form of excessive dripping from meat and meat products can represent a significant loss of weight from carcasses and cuts and may affect the yield and quality of processed meats, as well as being unattractive for consumers [1]. It has been proven that drip loss, as a measure of WHC, is one of the most important parameters of meat quality evaluation [2]. The amount of excessive drip loss affects both qualitative and quantitative aspects of a muscle, while the industry requires methods that can easily, and with a great precision, determine the WHC of meat and meat products [2].

The two most widely utilized gravimetric methods for measuring drip loss are the bag method, recognized internationally as the standard method, and the relatively new, but widely used in the industry, EZ-DripLoss method [3]. The EZ-DripLoss method was suggested by Rassmussen and Anderson [4] and is described in detail in the “Instruction manual for EZ-DripLoss” [5]. In brief, EZ-DripLoss is a method in which the meat is left suspended in a specialized EZ airtight container for a 24 h-long period to drip, with gravity as the only force exerted on the meat. Abundant research has reported that the EZ-DripLoss method has greater sensitivity, is easier to perform in a reproducible way, and uses less space than other conventional methods [2,6,7,8]. However, it must be taken into consideration that in relation to the well-standardized bag method, there is a broad diversity in procedures related to the EZ-DripLoss method.

According to the “Instruction manual for EZ-DripLoss” [5], drip loss is evaluated after a 1 d measurement interval, and the sample core fiber orientation is vertical. With porcine meat, Otto et al. [9] and Filho et al. [2] recommended using a 2 d measurement interval and a 45° fiber orientation for accuracy with the EZ-DripLoss method. Kilgannon et al. [10] suggested a 3 d measurement interval as ‘best practice’ to determine drip loss when using the EZ-DripLoss method for beef. Holman et al. [11] reported that fiber orientation (vertical vs. horizontal) did not affect EZ-DripLoss, and that longer measurement intervals (more than 4 d) are recommended for lamb meat. In addition to inconsistencies of used methodology, it is well known that the results of used drip loss methods are highly dependent on the area sampled, its weight, the fiber direction of the sample, and the storage period [2,11,12]. Furthermore, from the literature reports, it is obvious that there is a deficiency in our knowledge of the EZ-DripLoss method’s suitability for poultry meat analysis. Regarding the aforementioned, this study aimed to research the effect of different sample core diameters (Ø 10, 20, and 30 mm), and fiber orientations (vertical vs. horizontal) on the EZ-DripLoss in chicken breast meat measured across a period of five days.

## 2. Materials and Methods

The study was conducted in accordance with Croatian legislation (Animal Protection Act, Official Gazette 102/17; Regulation on the Protection of Animals Used for Scientific Purposes, Official Gazette 55/13), and was approved by the Bioethical Committee for the Protection and Welfare of Animals at the University of Zagreb, Faculty of Agriculture, Croatia (Class: 114-04/20-03/10; Ref. 251-71-29-02/19-20-2, 30-11-2020).

### 2.1. Animals and Muscle Sampling

A total of 40 chicken broilers from the line Ross 308 were used in the study. The animals were slaughtered at 35 d of age. After the slaughtering procedure and evisceration process, the carcasses were chilled at 4 °C for 24 h in a cold chamber.

The samples for the analysis were taken in the laboratory from the pectoralis muscle (PM) of each carcass at 24 h post-mortem. Chicken breast meat was manually trimmed of skin, visible fat, and connective tissue. In detail, two slices (diameter 30 mm; height 35 mm) were removed from the cranial side of the PM. From each cranial end of the slice, three cylindrical muscle cores were removed (from the lateral to the medial side) using EZ-DripLoss circular knives. The muscle cores were removed following the fiber orientation (being horizontal or vertical in relation to the circular knife), and having a 10, 20, and 30 mm diameter. Each individual meat sample (*n* = 240) was weighed, placed in a special EZ container (Danish Meat Research Institute, Taastrup, Denmark), and stored in a refrigerator at an average temperature of +3.5 °C (±0.80 s.d.). Sample cores were reweighed at daily intervals over a period of five days (in total: five measurement intervals). Drip loss assessment was performed after the aforementioned measurement intervals based on the change in sample weight, and was expressed as a percentage. Before each final weighing, the muscle surface samples were dabbed.

### 2.2. Statistical Analyses

Data was analyzed using the SAS/STAT software package version 9.4 [13]. Basic statistic parameters for EZ-DripLoss were calculated using the MEANS procedure, while the analysis of variance was performed by using MIXED procedures. The following mixed model was applied for the analysis of each type of fiber orientation (vertical vs. horizontal) of EZ-DripLoss:

*y_ijk_* = *C_i_* + *b_i_* (*x_ijk_*) + *s_j_* + *e_ijk_*,

where: *y_ijk_*—dependent variable, *C_i_*—fixed effect of core diameter (*i* = 10, 20, and 30 mm), *b_i_*—interaction of linear regression coefficient with effect of core diameter, *x_ijk_*—independent variable hour, *s_j_*—random effect of sample (*j* = 1, 2, 3, …, 40), and *e_ijk_*—random residual. For graphical interpretation of regression curves and confidential limits, the PLM procedure was used. To assess the difference between vertical and horizontal fiber orientations, they were included as a fixed effect in the aforementioned mixed model. Post hoc comparison among the least square means between fiber orientations (vertical vs. horizontal) and core diameters (10, 20, and 30 mm) was performed using a Bonferroni multiple test correction. The differences were considered to be significant if *p* < 0.05.

## 3. Results and Discussion

First of all, it is necessary to present that the fitted models had a high explanatory and predictive ability of the EZ-DripLoss in reference to fiber orientation. Prediction of EZ-DripLoss by using vertical fiber orientation provided the overall coefficient of determination (R^2^) of 0.82, whereas for horizontal fiber orientation R^2^ was 0.87.

### 3.1. The Influence of Sample Area on the EZ-DripLoss

**Figure 1:**
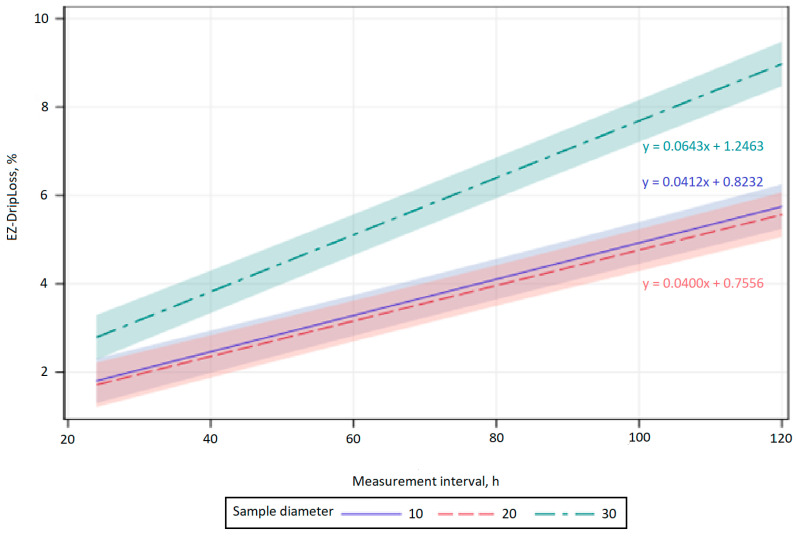
Predictions and confidence bands for a linear regression of measurement interval for EZ-DripLoss of chicken breast meat in relation to vertical fiber direction and core diameter (10, 20, and 30 mm).

**Figure 2:**
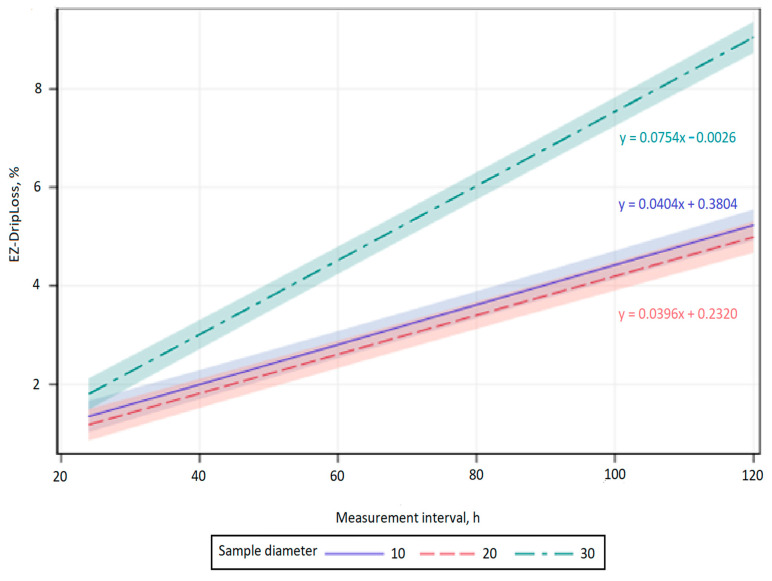
Predictions and confidence bands for a linear regression of measurement interval for EZ-DripLoss of chicken breast meat in relation to horizontal fiber direction and core diameter (10, 20, and 30 mm).

The influence of the area sampled on the EZ-DripLoss of chicken breast meat with respect to the vertical and horizontal fiber orientation and the measurement interval of 24, 48, 72, 96, and 120 h was statistically significant between samples with a diameter of 10 and 30 mm, and a diameter of 20 and 30 mm (*p* < 0.001; Figure 1 and Figure 2).

Differences between estimated linear regression coefficients of the EZ-DripLoss between samples with a diameter of 10 and 30 mm can be attributed to manipulative procedures when sampling, i.e., excluding samples of 10 mm with circular knife, and excluding samples of 30 mm from the containers prior to weighing. In addition, Holman et al. [11] pointed out that smaller samples also had a lower retention force, i.e., immobilization of retained water, which means that they displayed significantly more drip loss. With alpaca meat, Logan et al. [8] reported significant differences in drip loss determined by the bag method between samples of different weights. The authors found a drip loss of 4.04% in samples weighing 80 g, and 3.38% in samples weighing 60 g.

### 3.2. The Influence of Fiber Orientation on EZ-DripLoss

Of particular interest was the influence of the fiber orientation on the EZ-DripLoss of chicken breast meat. The results indicate that the fiber orientation significantly affects the EZ-DripLoss in chicken breast meat (*p* < 0.0001). It was found that the samples with vertical fiber orientation had a 0.50% greater drip loss compared to the samples with horizontal fiber orientation. This difference could be attributed to structural elements in muscle where ‘free water’ expelled from meat as drip loss is associated with vertical fiber direction and its movement in vertical samples is accelerated due to the greater gravimetric forces [2,11]. With lamb meat, however, Holman et al. [11] did not find a statistically significant difference in drip loss between the samples with vertical and horizontal fiber orientation during measurements at four 24 h intervals (24, 48, 72, and 96 h).

### 3.3. The Influence of Measurement Interval on EZ-DripLoss

The results of the present study indicate a significant and positive linear increase in EZ-DripLoss for the samples with horizontal and vertical fiber direction whose diameter was 10, 20, and 30 mm during all investigated measurement intervals (*p* < 0.001; Figure 1 and Figure 2). The estimated regression coefficient showed that drip loss for the samples with horizontal and vertical fiber direction whose diameter was 10 and 20 mm, significantly increased by 0.04% per hour. In addition, the estimated regression coefficient also showed that drip loss for the samples with vertical fiber direction whose diameter was 30 mm, significantly increased by 0.06% per hour, and for the horizontal fiber direction, significantly increased by 0.07% per hour. As already mentioned, an increase in drip loss over time is expected, as exudation is known to be a slow process that lasts for days, during which water expelled from the myofibrils accumulates in the muscle [14]. The results of the present study (Table 1 and Table 2) are in accordance with previous studies using the EZ-DripLoss method [2,11,15,16,17].

With pork meat, Correa et al. [15] found lower average drip losses within 24 h (3.54%) than within 48 h (4.66%). With chicken breast meat, Graberec et al. [16] also reported lower average drip losses within 24 h (2.38%) than within 48 h (2.74%). In a study on pork meat, Filho et al. [2] found average drip losses of 3.13% within 24 h and 5.19% within 48 h. With mutton, Kaić et al. [17] reported average drip losses of 0.65% within 24 h and 0.93% within 48 h. With lamb meat, Holman et al. [11] also reported that drip losses increased with the measurement interval (0.41% = 24 h; 0.67% = 48 h; 0.96% = 72 h; and 1.49% = 96 h).

## 4. Conclusions

The study indicates that the EZ-DripLoss in chicken breast meat is highly dependent on the area sampled, fiber orientation, and measurement interval. With respect to the sampled core diameters of 10, 20, and 30 mm, it could be suggested that samples of 20 mm are more suitable for EZ-DripLoss evaluation due to their lower drip losses, and greater effectiveness in sampling procedures. Greater drip losses in the samples with vertical fiber orientation suggest that horizontal fiber orientation should be used when chicken breast meat is evaluated using the EZ-DripLoss method. The measurement interval showed a positive linear relationship with drip loss, indicating that longer periods are needed for its stabilization in chicken breast meat and should be reported in studies that use the EZ-DripLoss method. Although the EZ-DripLoss method is considered to be simple and reproducible, it still has not been researched and standardized. Comparisons of the EZ-DripLoss results obtained with different sampling procedures should be performed with great caution.

## Figures and Tables

**Table 1:** Means ($\bar{x}$) with standard deviation (SD), coefficients of variation (CV), minimum (Min), and maximum (Max) for the drip loss of chicken breast meat in relation to vertical fiber direction, core diameter (10, 20, and 30 mm), and measurement interval (24, 48, 72, 96, and 120 h).

Core Diameter/Measurement Interval		*n*	$\bar{x}$	SD	CV, %	Min	Max
10 mm	24 h	40	1.81	0.958	52.77	0.23	3.83
	48 h	40	2.79	1.065	38.18	0.85	5.90
	72 h	40	3.77	1.358	36.04	1.60	7.72
	96 h	40	4.76	1.539	32.34	2.35	8.98
	120 h	40	5.75	1.760	30.59	3.10	11.02
20 mm	24 h	40	1.72	1.105	64.39	0.40	4.76
	48 h	40	2.70	1.176	43.52	0.90	5.45
	72 h	40	3.62	1.451	40.12	1.20	7.20
	96 h	40	4.59	1.664	36.26	1.95	8.86
	120 h	40	5.58	1.752	31.39	2.71	10.24
30 mm	24 h	40	2.87	2.336	81.45	0.00	9.04
	48 h	40	4.34	2.225	51.21	1.44	9.20
	72 h	40	5.74	2.360	41.13	1.44	11.82
	96 h	40	7.38	2.253	30.51	2.88	14.09
	120 h	40	9.07	2.450	27.00	4.32	16.36

**Table 2:** Means ($\bar{x}$) with standard deviation (SD), coefficients of variation (CV), minimum (Min), and maximum (Max) for the drip loss of chicken breast meat in relation to horizontal fiber direction, core diameter (10, 20, and 30 mm), and measurement interval (24, 48, 72, 96, and 120 h).

Core Diameter/Measurement Interval		*n*	$\bar{x}$	SD	CV, %	Min	Max
10 mm	24 h	40	1.34	0.596	44.49	0.39	2.89
	48 h	40	2.30	0.823	35.74	0.87	4.58
	72 h	40	3.34	1.042	31.25	1.50	6.33
	96 h	40	4.28	1.207	28.18	2.20	7.68
	120 h	40	5.20	1.360	26.14	2.91	9.03
20 mm	24 h	40	1.21	0.505	41.73	0.44	2.70
	48 h	40	2.08	0.640	30.72	1.13	3.66
	72 h	40	3.11	0.827	26.60	1.61	5.33
	96 h	40	4.05	0.946	23.38	2.40	6.10
	120 h	40	4.99	1.080	21.67	3.15	7.10
30 mm	24 h	40	1.98	1.163	58.82	0.00	5.69
	48 h	40	3.41	1.381	40.55	1.33	8.13
	72 h	40	5.41	1.328	24.54	2.86	11.38
	96 h	40	7.22	1.628	22.55	4.29	13.82
	120 h	40	9.12	1.828	20.04	6.43	16.26

## Data Availability

The data that support the findings of this study are available from the corresponding author, A.K., upon reasonable request.
